# Remarks on Abortion

**Published:** 1823-03

**Authors:** H. W. Ward

**Affiliations:** Surgeon.


### Art. VII.—*Remarks on Abortion.* By H. W. Ward, Esq. Surgeon.

If inquiries become interesting as the subject on which they are made are intricate, the following will, I think, be allowed to be one of this number; and I trust it may be the means of causing the further investigation of a subject of such importance. The cause of the death of the fœtus in utero, is a subject which has for many years occupied the talent of many able men, and it has been referred to a great variety of sources; but the practice which a removal of these causes would in theory seem to warrant has, more frequently than otherwise, proved ineffectual: but, in those cases where the cause of the accident is apparent, it may sometimes be prevented; and, from an attentive consideration of this point, it is that I am enabled to report the success of the mode of practice I have now to lay before the profession. And here, in justice to Dr. D. Stewart, an able obstetric practitioner and lecturer, I acknowledge that I was in a great measure led to this plan of proceeding, by learning from him the efficacy of opiate suppositories in preventing the death of the fœtus in utero, by allaying the irritation of the bowels, which he considered the cause of it.

It was an idea entertained by that able practitioner, the late Sir Richard Croft, that diarrhœa generally preceded or accompanied abortions; but, with deference to that gentleman's opinion, I think diarrhœa is frequently the immediate cause of it: and why I entertain such an opinion, I shall hereafter point out.—Some time ago I was called in to attend a patient, who was in the fifth month of pregnancy, but had no pains that seemed to warrant any idea of labour. I was informed by her attendants, she had never borne a living child, but had aborted three successive times, at this period of gestation; and, from the similarity of symptoms with which she was now attacked, they greatly feared a recurrence of the accident. I regretted that I had not been called in earlier, as a diarrhœa, which was always the most prominent symptom, had existed for four days. As my patient was extremely exhausted by the complaint, I ordered an aromatic cretaceous mixture, with opium; but, in less than eight hours, the fœtus was expelled, dead. The placenta was retained, as is common in such cases, longer than is usually the case in natural labours, and, after waiting an hour, I extracted it. No untoward symptom arose, and my patient recovered.

I was again called on to visit this patient, then also in the fifth month of pregnancy, and labouring under precisely the same train of symptoms as before detailed. As I considered it would be, perhaps, a more fortunate practice, I ordered a laxative draught with oleum ricini; which, however, produced no alteration in the nature or quantity of the alvine evacuations, and I again ordered the cretaceous mixture as before. Notwithstanding this, my patient still continued to labour under these symptoms. I then ordered the cretaceous mixture to be laid aside, and recommended the use of opiate suppositories, which I had hitherto known to be of great service in preventing similar accidents, arising from irritation in the bowels, which I was of opinion was the cause of all the mischief in this case. This plan, however, was attended with no good effect; and, fearing danger existed in delay, I also laid this aside, and wholly depended on large and repeated doses of opium, to be given with a view of allaying the irritation, and preventing a recurrence of the accident. This had the desired effect of subduing the complaint; my patient went through the usual period of utero-gestation, and was delivered of a living child. In the former illness to which I was called, I have no doubt that uterine contraction had begun, and therefore the opium was of no avail; but it must be evident that, if opium be freely given before such action be set up, it probably may—and I think I am right in saying, generally will,—be successful in preventing it. From what I have said in the preceding part of this paper, it will appear that, in the former illness, the diarrhœa did not abate, and abortion was the consequence; but in the latter, where it was checked by means of opium, my patient went through the usual period of utero-gestation, and was delivered of a living child; a circumstance which never before took place when the diarrhœa was unabated; and hence I think it reasonable to infer this was the cause of the former abortions.

I have said, where the cause of the accident is apparent, it may sometimes be prevented (for we know, if arising from a syphilitic taint, debility, or diseases of the uterus, it may be remedied,) by a removal of these causes: but, on the other hand, cases will occur where all our exertions will be foiled.

Among them may be classed those we sometimes find, where the uterus will not allow a sufficient distention to go through the full period of utero-gestation: others, where the uterine vessels do not supply a sufficient quantity of blood for such purpose. And it will also be readily admitted that, where one abortion has taken place, it is generally the precursor to another.

The principal motive I have had in view, has been to stimulate those members of the profession, whose obstetric practice is extensive, to a further inquiry into this important subject.

*Maidenhead; November,* 1822.

